# Effect of ZnO on Luminescence Performance of Terbium-Activated Zinc Borosilicate Glasses

**DOI:** 10.3390/ma17092154

**Published:** 2024-05-04

**Authors:** Sena Dayioglugil, Nuri Solak

**Affiliations:** Faculty of Chemical-Metallurgical Engineering, Istanbul Technical University, Istanbul 34469, Turkey; dayioglugil@itu.edu.tr

**Keywords:** zinc borosilicate glasses, luminescence, Tb^3+^, glass structure

## Abstract

In this study, terbium-doped ZnO-SiO_2_-B_2_O_3_-Na_2_O glasses were fabricated with the conventional melt-quenching method. The effect of altering the concentration of the host matrix on luminescence performance was investigated in terms of different ZnO/B_2_O_3_ and ZnO/SiO_2_ ratios. FT-IR results indicate that bridging oxygens (Bos) were converted to non-bridging oxygens (NBOs) with increments of ZnO. Furthermore, the emission intensity and luminescence lifetime of samples were influenced by the amount of ZnO; this was proven with photoluminescence spectra results. The maximum emission intensity was observed at a 1.1 ZnO/B_2_O_3_ ratio and a 0.8 ZnO/SiO_2_ ratio; however, the highest luminescence lifetime was observed at a 1.1 ZnO/SiO_2_ ratio. The emission intensity and luminescence lifetime of glass samples were improved by heat treatment as a result of the formation of willemite and zinc oxide phases. An increase in the ZnO/SiO_2_ ratio facilitated the formation of willemite and zinc oxide phases; therefore, crystallinity was directly related to the luminescence behavior of glass samples.

## 1. Introduction

Borosilicate glasses activated with rare earth ions have gained prominence in a wide range of luminescent applications, including solid-state lighting, laser technology, display panels, and optical devices, owing to their good optical transmission, thermal stability, chemical resistance, low manufacturing cost, and ease of fabrication into various forms. The high transmittance of borosilicate glasses allows the emission of light without creating a barrier in phosphorescence applications. B_2_O_3_ and SiO_2_ are excellent network formers that create bridging oxygen structures. However, the high phonon energies of these network formers lead to reduced luminescence efficiency as a result of non-radiative losses in the glass [1,2,3,4,5]. In order to prevent non-radiative energy losses and improve the luminescence performance of the glass system, modifier oxides can be added to borosilicate glasses. In particular, modifier oxides facilitate the formation of coordinated defects by disrupting Si-O-Si and B-O-B bonds. Zinc oxide is a promising modifier oxide that has low phonon energies and good rare earth ion solubility. ZnO has a wide band gap (eV); furthermore, the presence of ZnO in borosilicate glasses enhances light emission in the ultra-violet region [6,7,8]. ZnO acts as a modifier oxide by occupying the interstitial position in the glass network. This leads to a disruption of network connectivity, an alteration in bond formation, and the creation of non-bridging oxygens [4,9,10]. Moreover, the crystallization behavior of a glass matrix depends on the amount of ZnO. The formation of zinc oxide (ZnO) and willemite (Zn_2_SiO_4_) phases affects the optical properties of glass. Zn_2_SiO_4_ is recognized as a good candidate for hosting phosphors and optical materials thanks to its wide band gap, which allows for efficient light emission, chemical stability, and transparency in the UV-visible region [11,12,13]. The addition of an alkaline metal such as Na_2_O to the glass composition contributes to stabilization during melting by increasing its stiffness [14].

Prior works indicated that various rare earth (Tb^3+^, Sm^3+^, Pr^3+^, and Ce^3+^)- and transition metals (Mn^2+^, Cu^2+^, and Co^2+^)-activated zinc borosilicate glasses have been studied to investigate luminescence properties [15,16,17,18,19,20,21,22]. Furthermore, the effect of adding transition metals (Nb, Mn, V, and Co) on the crystallization behavior of zinc borosilicate glasses has been examined [18,22,23,24]. However, in those studies, the effect of substituting zinc oxide with silicon dioxide and boron oxide on the luminescence behavior of zinc borosilicate glasses was not investigated in detail.

For these reasons, ZnO-B_2_O_3_-SiO_2_-Na_2_O glasses appear to be an attractive host matrix for doped-with-rare-earth ions. The electron shell structure of all trivalent lanthanide ions is unfilled and consists of 4*f*^N^ electrons, where N ranges from 1 to 14. Optical transitions are related to 4*f* electrons [25]. Terbium ions are preferred as an activator material, as Tb^3+^ ions, which exhibit green emission even at low concentrations, have a high luminescence behavior in the visible band. The transition of Tb^3+^ ions arises from the Laporte-forbidden metastable state ^5^D_4_ to the lower states ^7^F_6 (_487 nm), ^7^F_5_ (542 nm), ^7^F_4_ (585 nm), and ^7^F_3_ (620 nm). Even small quantities of Tb^3+^ ions can be added to the host matrix; a strong green emission at 542 nm has been observed with the naked eye [26,27].

In this study, the dependence of phosphorescence properties on the composition of the glass matrix was investigated. The role of ZnO, B_2_O_3_, and SiO_2_ on luminescence phenomenon was explored via a ternary phase diagram of ZnO-B_2_O_3_-SiO_2_ glass systems with constant Na_2_O and Tb_2_O_3_. In addition, the effects of different heat treatment temperatures on crystallization behavior and of crystallization on luminescence behavior of glass systems were analyzed.

## 2. Materials and Methods

**Figure 1:**
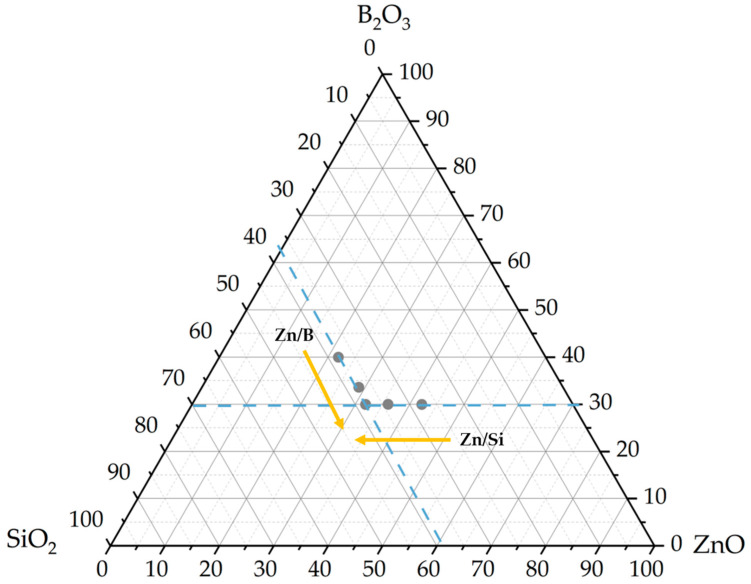
Phase diagram of glass samples.

Terbium-activated ZnO-B_2_O_3_-SiO_2_-10Na_2_O wt% glass samples were synthesized by the conventional melt-quenching technique (Table 1). Five terbium-activated glass samples were selected from the ternary phase diagram shown in Figure 1. In the first group of samples, the ratio of ZnO/B_2_O_3_ was varied, with a constant amount of SiO_2_. In the second group of samples, the ratio of ZnO/ SiO_2_ was varied, with a constant amount of B_2_O_3_. In all glass samples, the weight percentages of Tb_2_O_3_ and Na_2_O were constant.

Zinc oxide (ZnO), 99.5% (Merck, Darmstadt, Germany), boric acid (H_3_BO_3_), 99.5% (Merck, Darmstadt, Germany), sodium carbonate monohydrate (Na_2_CO_3_), 99.95% (Merck, Darmstadt, Germany), silicon (IV) oxide (SiO_2_), 99.9% (Alfa Aesar, Massachusetts, MA, USA) and terbium (III, IV) oxide (Tb_4_O_7_), 99.99% (Jiaton, Shanghai, China) were used as raw materials. Tb_2_O_3_ (0.35% mol) was added without disturbing the chemical composition of the host material. Raw materials were weighed in stoichiometric proportions and mixed in an agate mortar to provide homogeneity. Mixed batches were melted in platinum crucibles at a 1500 °C ambient atmosphere for 30 min. Melts were quenched to room temperature and poured into a stainless-steel plate. After obtaining transparent glass samples, heat treatment was applied at 650 °C, 750 °C, and 800 °C to investigate crystal structures; 650 °C and 750 °C were chosen for examination of phosphorescence properties of glass samples.

Glass structures were investigated using an X-ray diffractometer (XRD, Panalytical Aeris, Malvern, UK) using Co-Kα radiation. Powder and bulk forms of samples were used in XRD analysis. In order to determine glass transition and crystallization temperatures, differential scanning calorimeter (DSC; Netzsch STA 449F3 Jupiter, Selb, Germany) analysis was performed by heating the samples from room temperature to 1000 °C at a rate of 5 K/min under an argon atmosphere. A Fourier transform infrared spectrometer (FT-IR; Bruker Alpha-II, Billerica, MA, USA) was used in the spectral range of 400–4000 cm^−1^ at room temperature to investigate the presence of network structural and functional groups. Powder forms of glass samples were used in DSC and FT-IR analysis. The UV-visible absorption spectra analysis of glass samples was performed by an UV-VIS-NIR spectrometer (Agilent Cary 5000, Santa Clara, CA, USA) in ABS mode in the spectral range of 300–500 nm. The emission intensity and afterglow time of obtained glasses were determined using an Ocean Optics Flame spectrophotometer (Ocean Insight, Orlando, FL, USA) equipped with a 363 nm wavelength UV light lamp as an excitation source at room temperature.

## 3. Results and Discussion

### 3.1. XRD and DSC Analysis

XRD patterns of glass samples are given in Figure S1. As-cast transparent glass samples exhibited an amorphous structure without any peak formation.

**Figure 2:**
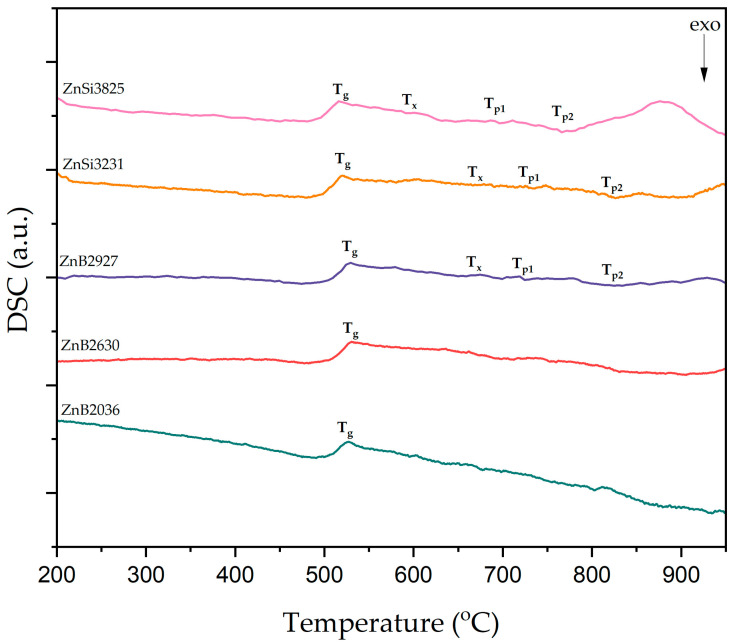
DSC curves of glass samples.

The characteristic glass temperatures of the samples were investigated by DSC analysis. Glass characteristic temperatures, detected as weak signals, were recorded at a heating rate of 5 K/min, and are shown in Figure 2. According to these DSC curves, endothermic peaks indicate glass transition temperatures (T_g_), while the first exothermic peaks correspond to the onset crystallization temperatures (T_x_) of glass samples. ZnSi glass samples exhibited two exothermic peaks that were related to crystallization temperatures (T_p_); however, crystallization peaks were not observed in ZnB2036 and ZnB2630 glass samples.

Characteristic glass temperatures (T_g_, T_x_, T_p1_, and T_p2_) are summarized in Table 2. DSC data results indicate that glass characteristic temperatures changed with the compositions of the glasses. The maximum amount of ZnO content was in the ZnSi3825 sample, which had the lowest Tg value. The decrease in the glass transition temperature may be explained by the strength of chemical bonds. The incorporation of ZnO into a host matrix facilitates the formation of non-bridging oxygens by breaking the bonds between oxygen ions and glass-forming ions [28,29,30,31].

**Figure 3:**
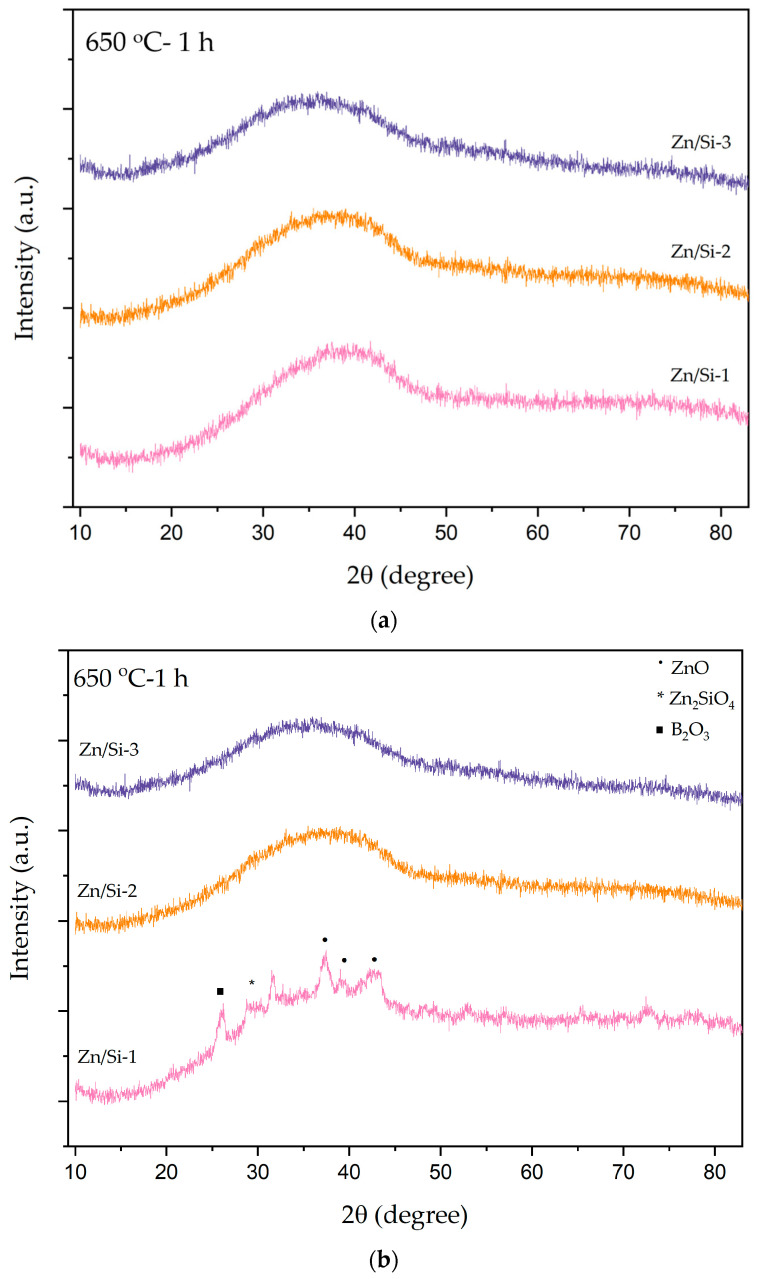
XRD patterns of ZnB2927, ZnSi3231, and ZnSi3825 glass samples after heat treatment at 650 °C for 1 h. (**a**) Powder samples and (**b**) bulk samples.

A heat treatment process was applied to ZnB2927, ZnSi3231, and ZnSi3825 glass samples at 650 °C, 750 °C, and 800 °C to determine structural changes. Figure 3 shows XRD patterns of both powder and bulk forms of samples at 650 °C for 1 h. Crystallization peaks for ZnO, Zn_2_SiO_4_, and B_2_O_3_ phases were detected in the ZnSi3825 bulk forms of samples; however, the powder forms of samples had amorphous structures. No crystallization peaks were revealed for ZnB2927 and ZnSi3231 samples, indicating the presence of amorphous structures. These two samples preserved the transparent appearance.

**Figure 4:**
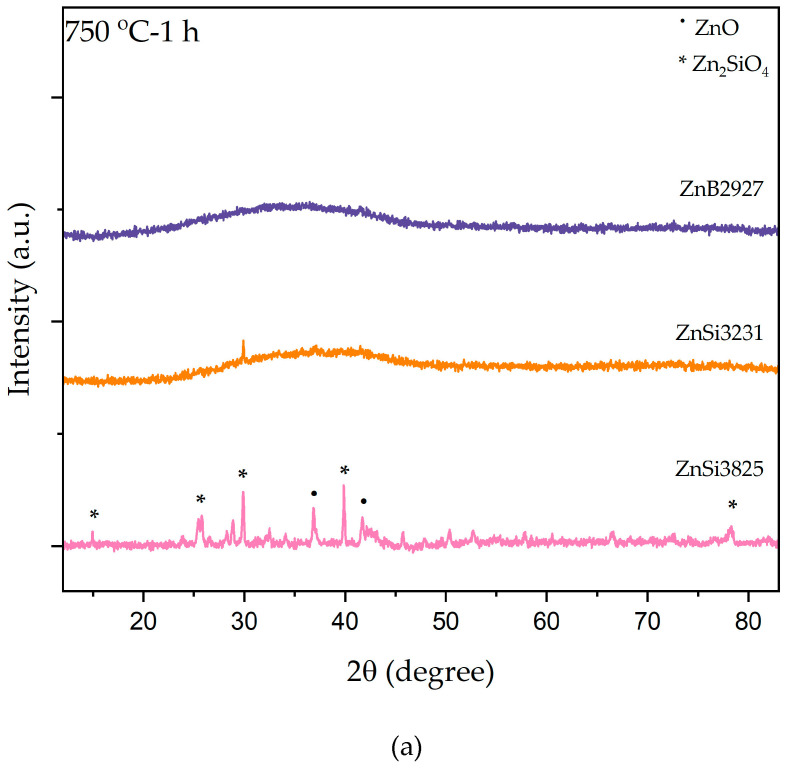

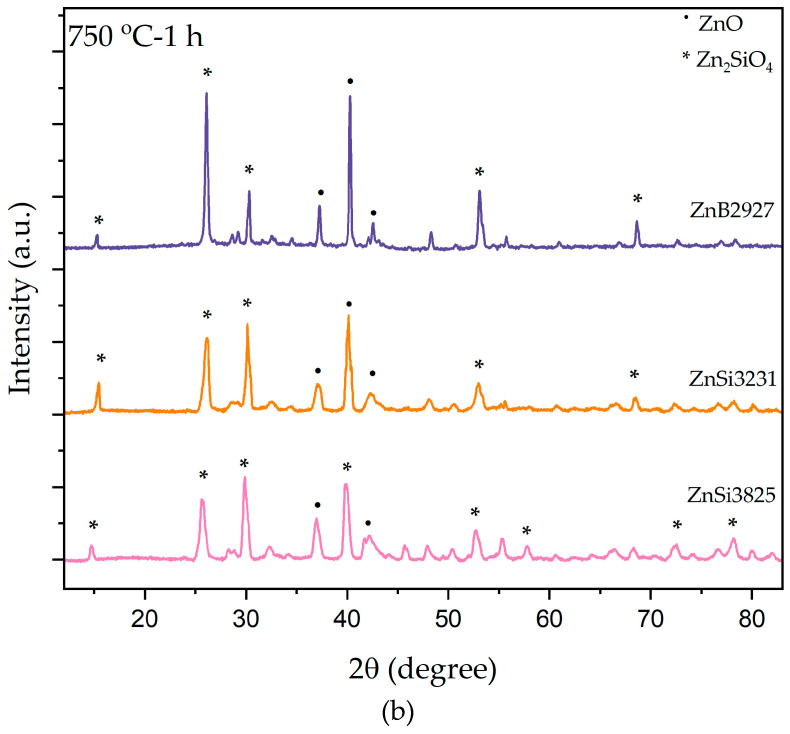
XRD patterns of ZnB2927, ZnSi3231, and ZnSi3825 glass samples after heat treatment at 750 °C for 1 h. (**a**) Powder samples and (**b**) bulk samples.

Figure 4 illustrates XRD patterns of both bulk and powder forms of samples at 750 °C for 1 h. As a result of heat treatment, ZnSi3825 bulk samples exhibited mainly opaque appearances, and ZnB2927 and ZnSi3231 bulk samples were opaque only on the surface. ZnO (zinc oxide) and Zn_2_SiO_4_ (willemite) phases were identified in the XRD patterns of powder and bulk forms of ZnSi3825. Although powder ZnB2927 and ZnSi3231 samples showed amorphous structures, surface crystallization was observed in these samples. ZnO and willemite phase peaks were present in the XRD pattern. When comparing XRD results at 750 °C, the formation of the willemite phase was more intense in the ZnSi3825 sample, which had the maximum amount of ZnO.

**Figure 5:**
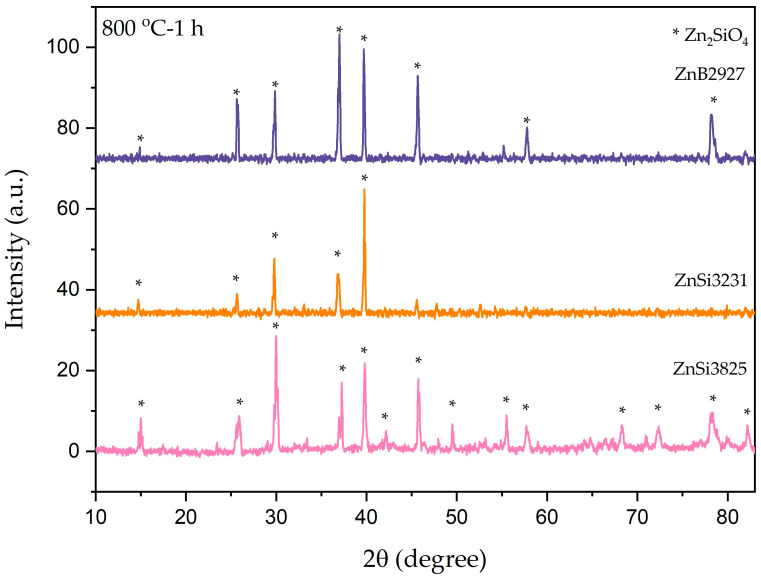
XRD patterns of ZnB2927, ZnSi3231, and ZnSi3825 glass powder samples after heat treatment at 800 °C for 1 h.

The transparency of the samples was lost as a result of heat treatment at 800 °C for 1 h. According to the XRD patterns of the samples, only the willemite phase was present, as shown in Figure 5. The conversion of ZnO phases to willemite phases occurred by heating samples to 800 °C.

Consequently, the degree of crystallization increased with the increments of the ZnO/SiO_2_ ratio. Crystallization behavior was related to the amount of ZnO (modifier oxide) in the host matrix. A higher amount of ZnO facilitated the formation of the willemite phase at lower temperatures [29,30,31,32,33].

### 3.2. FT-IR Analysis

**Figure 6:**
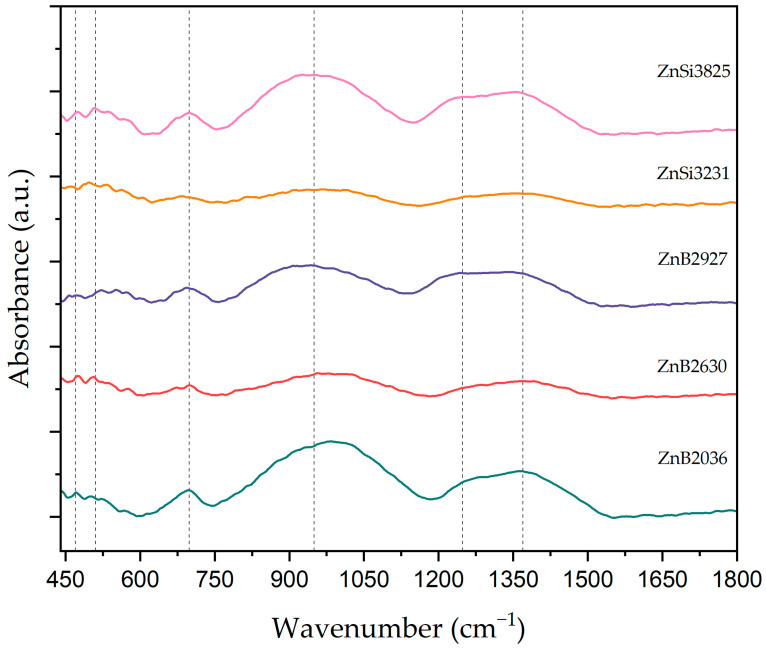
FT-IR spectra of glass samples.

FT-IR spectra were used to investigate network structural units present in ZnO-SiO_2_-B_2_O_3_-Na_2_O glasses. Figure 6 shows absorption results of glass samples in the 400–1800 cm^−1^ wavenumber region. Two regions exist in the FT-IR spectrum. One consists of the main characteristic absorption bands between 400 and 1500 cm^−1^, while the other region is weak absorption peaks between 1500 to 4000 cm^−1^. It can be seen in Figure 6 that all glass samples exhibited six absorption bands, and band positions of the samples are shown in Table 3. Bands that appeared at ~470 cm^−1^ and ~505 cm^−1^ were assigned to weak absorption peaks of O-B-O bending vibrations and symmetric stretching vibrations of ZnO_4_, respectively [34,35,36,37,38]. Bending vibrations of bridging oxygens between B-O-B bonds in BO_3_ units or Si-O-Si symmetric stretching of bridging oxygen between SiO_4_ tetrahedral units can be induced to absorption peaks at ~699 cm^−1^ [34,39]. Vibrational modes of tetrahedral BO_4_ groups may be observed in the spectral range of 952–975 cm^−1^ [36]. A weak shoulder at 1235–1270 cm^−1^ can be attributed to B–O stretching vibrations of (BO_3_) units with non-bridging oxygen atoms [40,41,42]. The broad band observed in the spectral range at 1356–1387 cm^−1^ may be assigned to the asymmetrical stretching vibration of B-O-B in BO_3_ [39,40,42]. The asymmetrical stretching vibration of B-O-B in the BO_3_ band shifted towards a lower wavenumber with an increase in the ZnO content in the host matrix.

In order to calculate the amount of non-bridging and bridging oxygen in the host matrix, deconvoluted FT-IR spectra of the samples were analyzed. FT-IR curves were deconvoluted to separate overlapping peaks to identify precise peak positions (PC) and area under the curve (A). Deconvoluted FT-IR spectra of the samples were obtained by the nonlinear curve fit module using the Gaussian function. The bands at ~699 cm^−1^, ~1270 cm^−1^, and 1370 cm^−1^ were related to bending vibrations of B-O-B bonds in BO_3_ units, B–O bond stretching vibrations of BO_3_ units, and asymmetrical stretching vibration of B-O-B in BO_3_ units, respectively. Furthermore, these vibrational modes assisted the formation of NBOs in the host matrix. Additionally, the band in the range of 900–1000 cm^−1^ was related to B–O bond stretching vibrations of BO_4_ units, which assisted the formation of BOs in the host matrix. These values were estimated from the area under the defined peaks and calculated using the following formulas [38,43,44,45].

(1) $$BO_{3}\;(N_{3})=\frac{A_{3}}{A_{3}+A_{4}}$$

(2) $$BO_{4}\;(N_{4})=\frac{A_{4}}{A_{3}+A_{4}}$$

**Figure 7:**
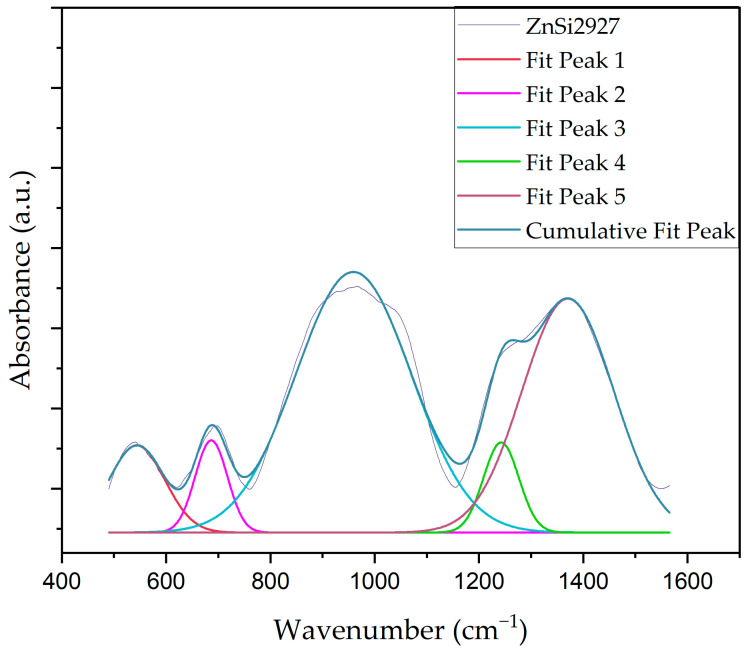
Deconvoluted FT-IR spectra of ZnB2927 glass sample.

In Equations (1) and (2), A_3_ and A_4_ are defined by the area under the peaks, which are related to BO_3_ and BO_4_ units, respectively. Deconvoluted FT-IR spectra of the ZnB2927 sample are given in Figure 7, and deconvolution peak center (cm^−1^) and area under the peaks are given in Table 4. According to Table 4, the N_3_ value increased with the addition of ZnO to the host matrix, which indicates that [BO_4_] units transformed into [BO_3_] units. The maximum amount of NBOs was obtained using the ZnSi2927 sample.

### 3.3. Photoluminescence Analysis

**Figure 8:**
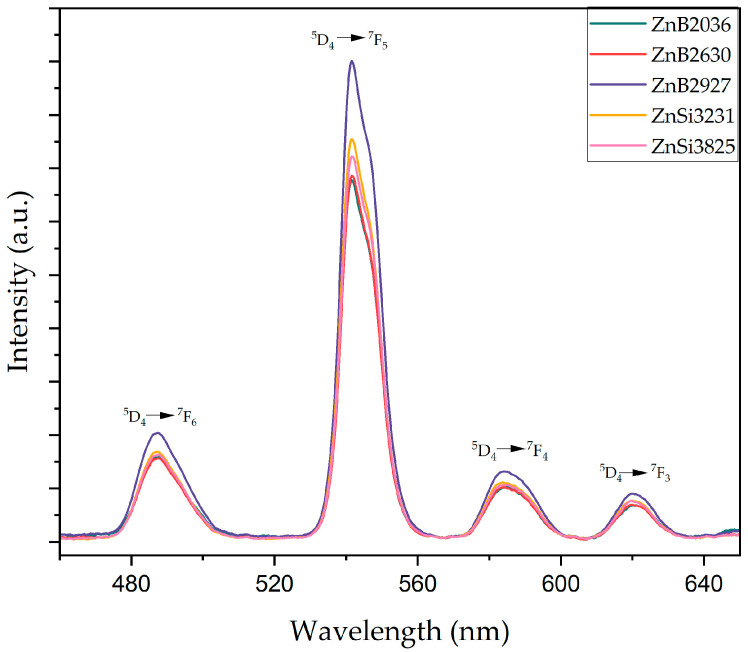
Phosphorescence spectra of glass samples after excitation with the 363 nm UV lamp.

UV–vis room temperature absorption spectra for Tb^3+^-doped zinc borosilicate glass samples are given in Figure S2 [46,47,48,49]. Phosphorescence properties of 0.35 mol% of Tb_2_O_3_-doped glasses were measured after samples were irradiated by a 363 nm wavelength UV light source for 5 min, as given in Figure 8. According to phosphorescence spectrophotometer results, four distinct emission peaks existed at 487 nm, 542 nm, 585 nm, and 620 nm, which arose from the ^5^D_4_→^7^F_6_, ^5^D_4_→^7^F_5_, ^5^D_4_→^7^F_4_, and ^5^D_4_→^7^F_3_ transitions of Tb^3+^ ions, respectively [15,46,50].

**Figure 9:**
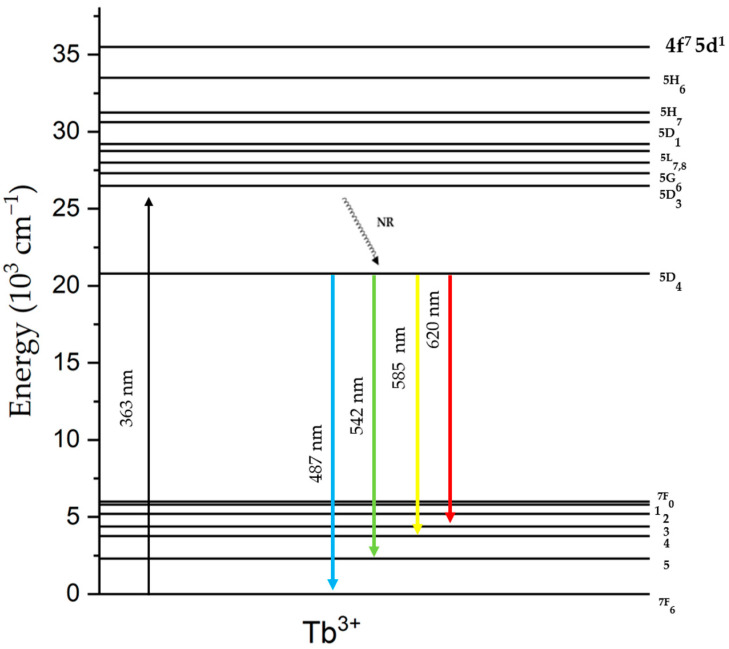
Energy level diagram of Tb^3+^-activated ZnO-B_2_O_3_-SiO_2-_Na_2_O glass samples.

Emission spectra can be described from a schematic energy level diagram of Tb^3+^ ions, as illustrated in Figure 9. Tb^3+^ ions were excited from the ground state ^7^F_6_ to the upper excited state ^5^D_3_ as a result of excitation with a 363 nm UV light. After stopping excitation, a non-radiative (NR) energy transition could occur due to cross relaxation caused by the interaction between Tb^3+^ ions in the glass matrix. Relaxation of Tb^3+^ ions to a metastable ^5^D_4_ state does not involve the emission of light. Consequently, radiative transition from the ^5^D_4_ state to a different ^7^F_j_ (j = 6,5,4,3) lower energy states leads to the emission of light. Glass samples had the highest intense peak centered at 542 nm, corresponding to the ^5^D_4_→^7^F_5_ transition [51,52,53].

Compositional changes could not influence the position of the emission peaks; however, they could affect the emission intensity of the peaks. The emission intensity of the first group of samples (with fixed SiO_2_ content) increased with the ZnO/B_2_O_3_ ratio. The main reason for the increment was the substitution of ZnO for B_2_O_3_ in the host matrix, which enabled the formation of NBOs. Furthermore, the concentration of the trap centers increased with the addition of ZnO, which acted as a UV emission center [54]. Therefore, the optimum amount of B_2_O_3_ was chosen at 30 wt%. In the second group of samples (with fixed 30 wt% B_2_O_3_), ZnO was replaced by SiO_2_. The emission intensity of the second group of samples was higher compared to that of the first group of samples. This behavior is explained by the non-bridging oxygens phenomenon. The higher amount of NBOs made it easier to excite samples due to weakened bonds [27,28,29,30,31,32,33,34,35,36,37,38,39,40,41,42,43,44,45,46,47,48,49,50,51,52,53,54,55]. Nevertheless, the emission intensity of samples decreased with an increase in ZnO/SiO_2_. The ZnSi3825 sample had the maximum amount of ZnO; however, its emission intensity was lowest due to zinc abnormality. As a result, the maximum emission intensity was observed in the ZnB2927 sample, which corresponded to the intersection of the two groups in the ternary phase diagram. The optimum ZnO/B_2_O_3_ and ZnO/SiO_2_ ratios were determined to be 1.1 and 0.8, respectively.

**Figure 10:**
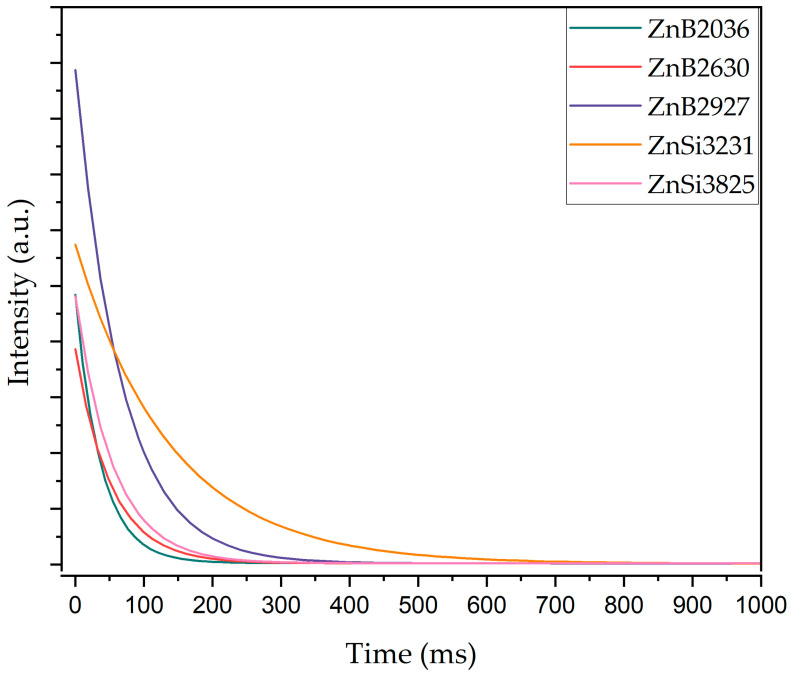
Decay curves of Tb^3+^-doped glass samples after excitation with the 363 nm UV lamp.

Figure 10 shows decay spectra analysis of glasses recorded for 542 nm emission under 363 nm excitation. The lifetimes of the samples were calculated by fitting experimental data to a non-exponential decay model. The non-exponential decay model is expressed as [56]

(3) $$I\left(t\right)=Ae^{\frac{-t}{\tau }}$$

In Equation (3), the intensity of samples at *t* = 0 is defined by *A*, *t* is time, and *τ* is the luminescence lifetime of samples. In Table 5, the luminescence lifetimes of samples are shown as a result of fitting of decay curves.

According to the decay curves, firstly, the luminescence lifetime increased from 51.7 to 139.9 ms with the increment in ZnO content; however, the lifetime of the ZnSi3825 sample decreased to 110.3 ms due to zinc abnormality. Consequently, the ZnSi3231 sample exhibited the maximum luminescence lifetime.

**Figure 11:**
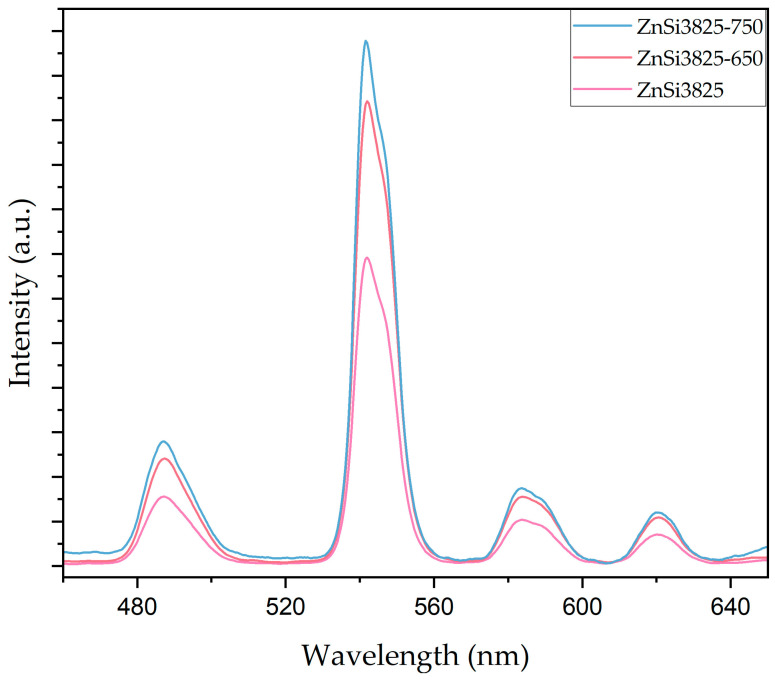
Phosphorescence spectra of untreated and heat-treated (at 650 °C and at 750 °C/1 h) ZnSi3825 glass samples after excitation with the 363 nm UV lamp.

After heat treatment at 650 and 750 °C for 1 h, photoluminescence spectra measurements were taken to investigate the effect of willemite and zinc oxide phases on luminescence behavior. Figure 11 illustrates phosphorescence spectra results for ZnSi3825 glass and heat-treated glass samples. Wavelength position did not change with the heat treatment, while the emission intensity of the samples increased. According to XRD patterns of the samples, the increase in concentration of ZnO content facilitated the formation of willemite and zinc oxide phases. Willemite and zinc oxide phases act as a UV emission center; therefore, the emission intensity of heat-treated samples was higher compared with that of the as-cast transparent samples [17,57].

**Figure 12:**
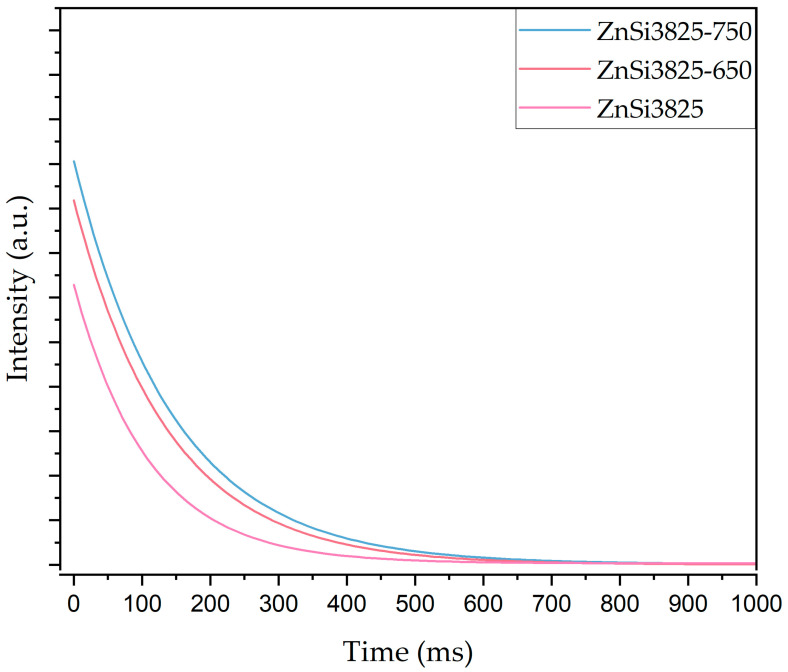
Decay curves of untreated and heat-treated (at 650 °C and 750 °C /1 h) ZnSi3825 glass samples after excitation with the 363 nm UV lamp.

Decay spectra analysis of glasses and heat-treated glasses samples were recorded at 542 nm emission under 363 nm excitation. Decay curves and luminescence lifetimes of glass samples are given in Figure 12 and Table 6, respectively. Luminescence lifetimes of heat-treated samples at 650 and 750 °C indicate that the presence of willemite and zinc oxide phases provided an increase in the afterglow time of samples. As a result, both emission intensity and lifetime of the samples improved with the crystallinity of willemite and zinc oxide phases.

## 4. Conclusions

Different compositions of ZnO-SiO_2_-B_2_O_3_-Na_2_O glasses with Tb^3+^ ions were prepared by the melt-quenching method to understand the influence of composition on luminescence behavior. Glass transition temperatures and crystallization temperatures decreased with an increase in the ZnO/B_2_O_3_ and ZnO/SiO_2_ ratio. Glass characteristic temperatures depend on the formation of non-bridging oxygens, which increase with the incorporation of ZnO into the glass matrix. FT-IR results confirm that the amount of NBOs increased with an increment of ZnO. Willemite and zinc oxide phases were observed in the heat-treated glass samples by XRD analysis. These phases were formed at lower temperatures in the ZnSi3825 sample, which had a 1.5 ZnO/SiO_2_ ratio.

Photoluminescence spectra results show the characteristic emission transition of Tb^+3^ ions under 363 nm excitation. The maximum emission intensity arose from the ^5^D_4_→^7^F_5_ transition at a 542 nm wavelength. The highest emission intensity was observed at 0.8 ZnO/SiO_2_ and 1.1 ZnO/B_2_O_3_ ratios; however, the highest luminescence lifetime was observed at a 1.1 ZnO/SiO_2_ ratio.

The increase in the ZnO/SiO_2_ ratio facilitated the formation of a willemite phase at lower temperatures. The presence of willemite and zinc oxide phases directly improved the phosphorescence properties of glass samples by creating a UV emission center; therefore, the emission intensity and lifetime of glass samples increased with heat treatment at higher temperatures.

## Figures and Tables

**Table 1:** Composition of glass samples doped with Tb^3+^.

ID		Batch Composition wt%					
	ZnO	B_2_O_3_	SiO_2_	Na_2_O	Tb_2_O_3_	ZnO/B_2_O_3_	ZnO/SiO_2_
ZnB2036	20	36	34	10	0.35	0.5	
ZnB2630	26	30	34	10	0.35	0.9	
ZnB2927	29	27	34	10	0.35	1.1	0.8
ZnSi3231	32	27	31	10	0.35		1.1
ZnSi3825	38	27	25	10	0.35		1.5

**Table 2:** Characteristic glass temperatures (T_g_, T_x_, T_p1_ and T_p2_) of samples calculated from DSC data.

	T_g_ (°C)	T_x_ (°C)	T_p1_ (°C)	T_p2_ (°C)
ZnB2036	526	-	-	-
ZnB2630	529	-	-	-
ZnB2927	527	674	724	824
ZnSi3231	519	685	733	826
ZnSi3825	517	601	696	766

**Table 3:** FT-IR band positions and assignments of vibrational mode.

Band Position (cm^−1^)	Assignment of Vibrational Mode	Ref.
~470	O-B-O bending vibrations/Silicate network Si–O–Si and O–Si–O bending mode	[34,35,36,37,38]
482–544	Symmetric stretching vibrations of ZnO_4_	[38]
~699	Bending vibrations of B-O-B bonds in BO_3_ units/ Si-O-Si symmetric stretching of SiO_4_ units	[34,39]
952–975	B–O bond stretching vibrations of BO_4_ units	[36,39]
1235–1270	B–O bond stretching vibrations of BO_3_ units	[40,41,42]
1356–1387	Asymmetrical stretching vibration of B-O-B in BO_3_ units	[39,40,42]

**Table 4:** Deconvolution peak center, area, BO_3_, BO4, N_3_, and N_4_ values of glass samples.

Sample	Peak Center (PC, cm^−1^), Area (A)						BO_3_	BO_4_	N_3_	N_4_
ZnB2036	PC	482	682	975	1270	1387				
	A	2.73588	0.98889	12.15383	0.66342	5.67783	7.33014	12.15383	0.38	0.62
ZnB2630	PC	538	674	970	1256	1381				
	A	0.34655	0.28086	2.53934	0.18676	1.47003	1.93765	2.53934	0.43	0.57
ZnB2927	PC	544	687	959	1243	1371				
	A	0.72837	0.44748	4.52	0.48003	3.21731	4.14482	4.52014	0.49	0.52
ZnSi3231	PC	541	685	957	1251	1367				
	A	0.64372	0.45881	4.88323	0.36963	2.76885	3.59729	4.88323	0.42	0.58
ZnSi3825	PC	544	693	952	1235	1356				
	A	1.80044	1.15739	12.73896	1.02464	7.43427	9.6163	12.73896	0.43	0.57

**Table 5:** Luminescence lifetimes (ms) of glass samples.

Sample	Lifetime of Glass (ms)
ZnB2036	37.3
ZnB2630	51.7
ZnB2927	67.0
ZnSi3231	139.9
ZnSi3825	110.3

**Table 6:** Luminescence lifetimes (ms) of glass and heat-treated samples.

	Lifetime (ms)		
Sample	Glass	Heat-Treated Samples at 650 °C/1 h	Heat-Treated Samples at 750 °C/1 h
ZnSi3825	110.3	138.0	145.6

## Data Availability

The data presented in this study are available on request from the corresponding author.

## Supplementary Materials

The following supporting information can be download at: https://www.mdpi.com/article/10.3390/ma17092154/s1, Figure S1. XRD patterns of as-cast transparent glass samples. Figure S2. Absorption spectra of Tb^3+^-doped glass samples in the UV-Vis region.
