# Supplementary material for: Effect of ZnO on Luminescence Performance of Terbium-Activated Zinc Borosilicate Glasses
Source: Materials (Basel). 2024 May 4;17(9):2154. doi: 10.3390/ma17092154 (PMC11084722; doi:10.3390/ma17092154)
Supplement: Supplementary file 1 [file materials-17-02154-s001.zip › materials-2972642-supplementary.pdf]

materials

Supplementary Materials

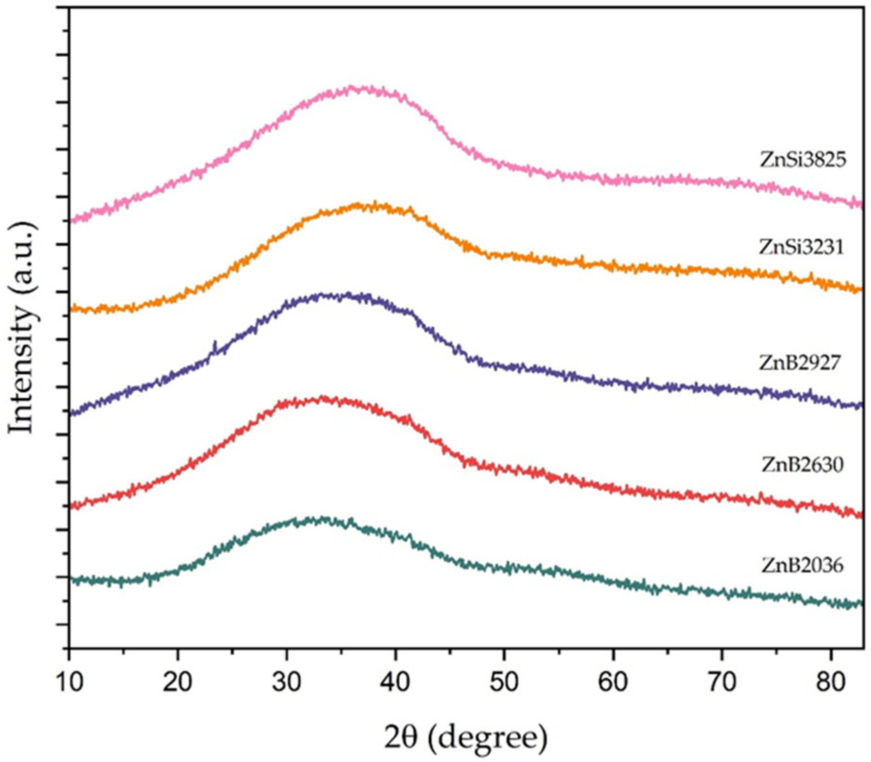

Figure S1. XRD patterns of as-cast transparent glass samples.

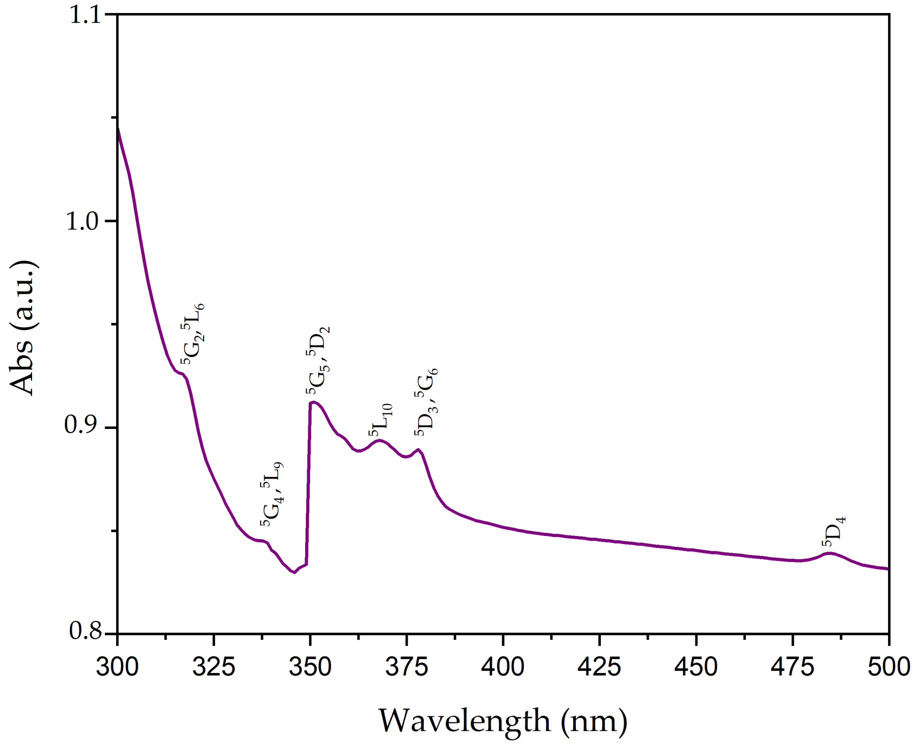

Figure S2. Absorption spectra of Tb^{3+}-doped glass samples in the UV-Vis region.

Figure S2 shows the UV–vis room temperature absorption spectra for all Tb^{3+}-doped zinc borosilicate glass samples. Six absorption peaks, at 317 nm, 338 nm, 351 nm, 368 nm, 378 nm, and 487 nm, were associated with the transition from the $^7F_6$ ground state to the higher excited states of ($^5G_2$,$^5L_6$), ($^5G_4$,$^5L_9$), ($^5G_5$,$^5D_2$), $^5L_{10,}$ ($^5D_{3,}$ $^5G_6$), and $^5D_4$, respectively [46–49].
